# Patient safety incident reports related to traditional Japanese Kampo medicines: medication errors and adverse drug events in a university hospital for a ten-year period

**DOI:** 10.1186/s12906-017-2051-2

**Published:** 2017-12-21

**Authors:** Yutaka Shimada, Makoto Fujimoto, Tatsuya Nogami, Hidetoshi Watari, Hideyuki Kitahara, Hiroki Misawa, Yoshiyuki Kimbara

**Affiliations:** Department of Japanese Oriental Medicine, Graduate School of Medicine and Pharmaceutical Sciences, University of Toyama, 2630 Sugitani, Toyama, 930-0194 Japan

**Keywords:** Adverse drug event, Drug-induced interstitial pneumonia, Kampo medicine, Medication error, Patient safety incident report, Scutellariae radix

## Abstract

**Background:**

Kampo medicine is traditional Japanese medicine, which originated in ancient traditional Chinese medicine, but was introduced and developed uniquely in Japan. Today, Kampo medicines are integrated into the Japanese national health care system. Incident reporting systems are currently being widely used to collect information about patient safety incidents that occur in hospitals. However, no investigations have been conducted regarding patient safety incident reports related to Kampo medicines. The aim of this study was to survey and analyse incident reports related to Kampo medicines in a Japanese university hospital to improve future patient safety.

**Methods:**

We selected incident reports related to Kampo medicines filed in Toyama University Hospital from May 2007 to April 2017, and investigated them in terms of medication errors and adverse drug events.

**Results:**

Out of 21,324 total incident reports filed in the 10-year survey period, we discovered 108 Kampo medicine-related incident reports. However, five cases were redundantly reported; thus, the number of actual incidents was 103. Of those, 99 incidents were classified as medication errors (77 administration errors, 15 dispensing errors, and 7 prescribing errors), and four were adverse drug events, namely Kampo medicine-induced interstitial pneumonia. The Kampo medicine (crude drug) that was thought to induce interstitial pneumonia in all four cases was Scutellariae Radix, which is consistent with past reports. According to the incident severity classification system recommended by the National University Hospital Council of Japan, of the 99 medication errors, 10 incidents were classified as level 0 (an error occurred, but the patient was not affected) and 89 incidents were level 1 (an error occurred that affected the patient, but did not cause harm). Of the four adverse drug events, two incidents were classified as level 2 (patient was transiently harmed, but required no treatment), and two incidents were level 3b (patient was transiently harmed and required substantial treatment).

**Conclusions:**

There are many patient safety issues related to Kampo medicines. Patient safety awareness should be raised to prevent medication errors, especially administration errors, and adverse drug events in Kampo medicine.

## Background

Reducing medical errors is a great international concern. In 2004, the World Alliance for Patient Safety was launched by the World Health Organization (WHO) to facilitate efforts by all Member States to make health care safer [1]. In 2005, the draft guidelines were developed, which introduced adverse event reporting to improve the safety of patient care [2]. The WHO is currently leading a global drive to build on patient safety education. A multi-professional edition of the Patient Safety Curriculum Guide was developed to assist effective capacity building in patient safety education [3].

Currently, incident reporting systems are widely used to collect information about patient safety incidents that occur in hospitals. Patient injuries, sometimes referred to as adverse events, are reported along with near-misses and equipment failures. In Japan, hospitals are mandated by the Ministry of Health, Labour and Welfare to have internal reporting systems. Any hospital or health facility can voluntarily report to an accreditation body, but there is a mandatory requirement to report to the Japan Council for Quality Health Care, which implemented a national reporting system in 2004. Reporting to this system is only mandatory for teaching hospitals, and voluntary for others. Information is reported electronically. The Japan Council for Quality Health Care analyses the facts and circumstances of the incident and provides feedback to the reporting entities. The data are classified and summary results are disseminated to health-care providers and to the public. Cases deemed particularly important are evaluated individually. Otherwise, reports are aggregated for statistical analysis [2, 4]. In 2015, the total number of medical institutions (hospitals) that reported medical adverse events was 1018; of those, 275 were teaching hospitals (i.e., having mandatory reporting), and 743 were non-teaching hospitals (i.e., having voluntary reporting) [4].

Traditional medicine has a long history of use in health maintenance, and disease prevention and treatment. In some countries, the majority of the population uses traditional medicine as primary care. In other countries, the use of traditional medicine has been more popular as an alternative or complement to Western medicine, and traditional or non-conventional medicine may be termed complementary or alternative medicine.

One of the three strategic objectives of the WHO Traditional Medicine Strategy 2014–2023 is to strengthen quality assurance, safety, proper use, and effectiveness of traditional and complementary medicine by regulating traditional and complementary medicine products, practices, and practitioners [5]. Meanwhile, one of the five strategic objectives of the Regional Strategy for Traditional Medicine in the Western Pacific (2011–2020) is to promote safe and effective use of traditional medicine [6].

Kampo medicine is traditional Japanese medicine, which originated in ancient traditional Chinese medicine, but was introduced and developed uniquely in Japan. Today, traditional Japanese medicines, Kampo medicines, are integrated into the Japanese national health care system; 148 Kampo extract formulations and 187 types of crude drugs (239 including crude drug powders) are approved by the Ministry of Health, Labour and Welfare and used under the national health insurance program [7, 8]. In contrast to other jurisdictions, Kampo medicines are both strictly standardised and regulated in Japan. However, no investigations have been conducted regarding patient safety incident reports related to Kampo medicines. Therefore, the aim of this study was to survey and analyse incident reports related to Kampo medicines in a Japanese university hospital to improve future patient safety.

## Methods

### Toyama university hospital and the Department of Japanese oriental medicine

The Toyama University Hospital is one of the Japanese National University Hospitals. According to data obtained from the hospital on 1 May 2016 [9], it has 612 patient beds (including 569 general and 43 psychiatric beds), and 372 physicians (including 34 residents), 699 nurses, 34 pharmacists, 120 medical technology staff, and 68 administrative staff working at the hospital. In the fiscal year ending in March 2016, the Toyama University Hospital served a total of 298,437 outpatients and 189,717 inpatients [9]. The Department of Japanese Oriental Medicine is one of the clinical departments in the Toyama University Hospital, providing medical care by integrating conventional and Kampo medicine; it served a total of 8752 outpatients and 900 inpatients during the aforementioned period [9].

### The patient safety incident reporting system

**Fig. 1:**
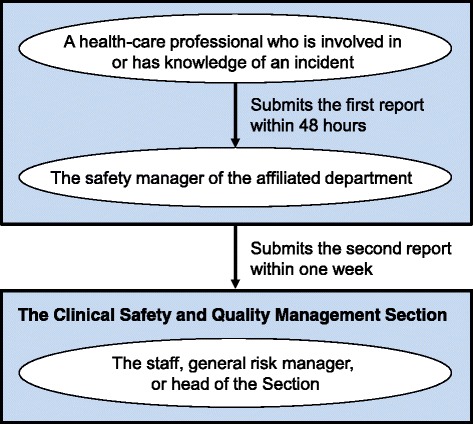
Flow chart of the incident reporting system

In the Toyama University Hospital, the Clinical Safety and Quality Management Section oversees patient safety affairs and is responsible for the incident reporting system. Reporting to the patient safety incident reporting system is mandatory for health-care professionals in the hospital. A health-care professional who is involved in or has knowledge of an incident submits the first report to the safety manager of their affiliated department within 48 h. The safety manager reviews the report, discusses countermeasures with the department’s staff, and submits the second report (within 1 week) to the Clinical Safety and Quality Management Section that includes ideas for prevention of recurrence. The staff subsequently reviews and investigates the report, and may request resubmission if necessary. A flow chart illustrating the incident reporting system in the Toyama University Hospital is provided in Fig. 1. This procedure was paper-based when implemented in 2004, but has been web-based since 2005, and monthly reports consisting of brief summaries of individual incident reports have been released within the hospital since May 2007.

In the case of a major incident, which is considered equivalent to or greater than level 3b (i.e., the patient was transiently harmed and needed substantial treatment) according to the incident severity classification system [10], the health-care professional who is involved in or has knowledge of the incident prioritizes treatment, then immediately informs the safety manager and head of their department. The involved department immediately (within 1 h) informs the general risk manager, the head of the Clinical Safety and Quality Management Section, or the director of the hospital.

### Incident severity classification

In Japanese hospitals, a classification system recommended by the National University Hospital Council of Japan is widely used to evaluate incident severity as part of the incident reporting system (Table 1) [10]. This incident severity classification is used in the Toyama University Hospital.

**Table 1:** Incident severity classification system recommended by the National University Hospital Council of Japan

Level	Continuity of injury	Severity of injury	Outcome/Treatment of injury
Level 5	Death	–	Death (excluding those due to the natural course of the underlying disease)
Level 4b	Permanent	Moderate–severe	Permanent disability or subsequent complication remained, accompanied with significant dysfunction or an aesthetic problem
Level 4a	Permanent	Mild–moderate	Permanent disability or subsequent complication remained, but was not accompanied by significant dysfunction or an aesthetic problem
Level 3b	Transient	Severe	Substantial treatment was required (significant change in vital signs, use of artificial respirator, surgery, prolongation of hospitalization, hospitalization, fracture, etc.)
Level 3a	Transient	Moderate	Simple treatment was required (disinfection, poultice, skin suture, administration of analgesics, etc.)
Level 2	Transient	Mild	Treatment was not necessary (mild change in vital signs, need for increased patient observation, examination for confirmation of safety, etc.)
Level 1	None	–	There was no harm to the patient (but there was a possibility of some influence)
Level 0	–	–	Error or trouble with a pharmaceutical or medical device was found, but did not affect the patient

### Medication errors and adverse drug events

According to the WHO’s concept, a medication error is defined as any preventable event that may cause or lead to inappropriate medication use or patient harm while the medication is in the control of the health-care professional, patient, or consumer [3]. Such events may be related to professional practice, health-care products, procedures, or systems. There are a number of distinct steps in medication use: prescribing, dispensing, administering, and monitoring are the main four. Physicians, pharmacists, other health-care professionals, and patients all play major roles in this process. For example, in the hospital setting, when a physician prescribes a medication, a pharmacist will then dispense the medication, and a nurse will administer it.

Additionally, the WHO defines an adverse drug event as an adverse event involving medication that may (e.g., the result of an error) or may not be (e.g., an unexpected allergic reaction in a patient taking a medication for the first time) preventable [3]. Furthermore, an adverse drug reaction is defined by the WHO as any response to a medication that is noxious and unintended, including injuries that are judged to be caused by drugs, but excluding drug-related injuries that result from error.

### Study design

We surveyed the monthly incident reports submitted between May 2007 and April 2017 in the Toyama University Hospital, selected all incidents related to Kampo medicines, and collected necessary information from the corresponding web-based original incident reports. The collected data included the date of the incident, the ward or department where the incident occurred, the health-care profession, years of experience and affiliated department of the reporter and person involved in the incident, information regarding the patient (outpatient or inpatient), incident details, classification of the incident (i.e., the step/stage at which the medication error [prescribing error, dispensing error, or administration error] or adverse drug event occurred), and the incident severity classification. The study’s design was approved by the Ethics Committee of the University of Toyama.

## Results

### Patients and incident reports in Toyama University hospital

The annual numbers of outpatients and inpatients, and the numbers of all incident reports and those related to Kampo medicines filed in the Toyama University Hospital and the Department of Japanese Oriental Medicine from May 2007 to April 2017 are shown in Table 2. The total number of incident reports filed in the Toyama University Hospital during the survey period was 21,324, while the number related to Kampo medicines was 108; the ratio of the latter to the former was 0.51%.

**Table 2:** The numbers of patients and incident reports filed from May 2007–April 2017

	2007^a^	2008	2009	2010	2011	2012	2013	2014	2015	2016	2017^b^	Total
N of OPs in TUH	189,546	283,428	293,205	297,022	302,328	305,478	304,136	301,813	296,167	297,475	98,655	2,969,253
N of OPs in DJOM (%)	8780 (4.63%)	12,642 (4.46%)	12,416 (4.23%)	12,018 (4.05%)	11,251 (3.72%)	11,329 (3.71%)	10,203 (3.35%)	9233 (3.06%)	8766 (2.96%)	8206 (2.76%)	2644 (2.68%)	107,488 (3.62%)
N of IPs in TUH	131,689	195,856	191,027	188,296	188,342	175,766	172,406	187,143	188,007	185,655	60,356	1,864,543
N of IPs in DJOM (%)	1628 (1.24%)	1887 (0.96%)	1691 (0.89%)	1794 (0.95%)	1677 (0.89%)	1207 (0.69%)	1178 (0.68%)	884 (0.47%)	946 (0.50%)	723 (0.39%)	115 (0.19%)	13,730 (0.74%)
N of IRs in TUH	1303	2043	2206	2101	2192	2057	2077	2366	2089	2175	715	21,324
N of IRs related to KMs in TUH(%)	14 (1.07%)	19 (0.93%)	14 (0.63%)	16 (0.76%)	12 (0.55%)	8 (0.39%)	7 (0.34%)	9 (0.38%)	4 (0.19%)	5 (0.23%)	0 (0.00%)	108 (0.51%)

*DJOM* Department of Japanese Oriental Medicine, *IPs* Inpatients, *IRs* Incident reports, *KMs* Kampo medicines, *N* Number, *OPs* Outpatients, *TUH* Toyama University Hospital

^a^Data for 8 months, from May to December 2007. ^b^Data for 4 months, from January to April 2017

### Incident reports related to Kampo medicines

Nurses, pharmacists, and physicians submitted 84 (77.8%), 15 (13.9%), and 9 (8.3%) incident reports, respectively, of the total 108 Kampo medicine-related reports during the 10-year survey period. Of the nine incident reports filed by physicians, the Department of Japanese Oriental Medicine physicians and other department physicians submitted six and three reports, respectively.

### Incidents related to Kampo medicines

Of the 108 Kampo medicine-related incident reports, five cases were reported redundantly (i.e., duplicate reports were submitted for the same incident); thus, there were 103 actual incidents. Regarding the professions of those involved in these incidents, 78 (75.7%), 15 (14.6%), and 10 (9.7%) were nurses, pharmacists, and physicians, respectively. Eighty-one (78.6%) incidents occurred on the hospital wards, 15 (14.6%) in the pharmaceutical department, and seven (6.8%) in outpatient clinics. Furthermore, 95 (92.2%) incidents were reported by the same individual that was involved in the incident (i.e., “identical person”), while eight (7.8%) were reported by a non-involved individual (i.e., “different person”). Meanwhile, with regard to the patients involved in the incidents, 92 (89.3%) were inpatients and 11 (10.7%) were outpatients.

**Fig. 2:**
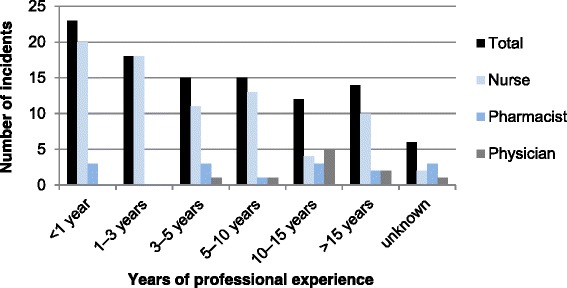
Years of professional experience of the persons involved in the Kampo medicine-related incidents

**Fig. 3:**
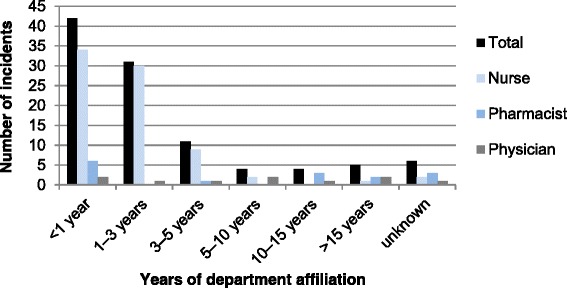
Years of department affiliation of the persons involved in Kampo medicine-related incidents

The distribution of the professional experience (in years) of the persons involved in the total 103 incidents, by their respective profession, is shown in Fig. 2. Similarly, the distribution of the department affiliation (in years) of the persons involved in the incidents is shown in Fig. 3.

### Medication errors and adverse drug events related to Kampo medicines

**Fig. 4:**
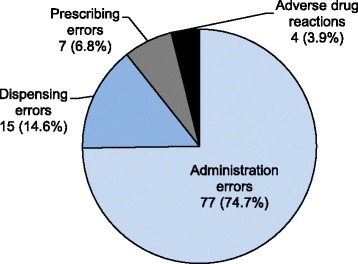
Medication errors (administration, dispensing, and prescribing) and adverse drug reactions in the Kampo medicine-related incidents

Of the 103 incidents, 99 (96.1%) were medication errors and four (3.9%) were adverse drug events (all adverse drug reactions). Of the medication errors, 77 (74.7%) were administration errors, 15 (14.6%) were dispensing errors, and seven (6.8%) were prescribing errors (Fig. 4). A more detailed classification of the medication errors and adverse drug events is presented in Table 3. All four adverse drug events were cases of Kampo medicine-induced interstitial pneumonia.

**Table 3:** Kampo medicine-related medication errors and adverse drug events from May 2007–April 2017

Medication errors	99
Administration errors	77
No administration	22
Administration not on time	22
Administration of the wrong dose	7
Administration of the wrong medicine	4
Administration to the wrong patient	8
Error in preparation for administration	6
Failure to confirm that medicine was taken	4
No administration when patient was staying outside the hospital	4
Dispensing errors	15
Dispensing the wrong medicine	8
Dispensing the wrong dose	1
Failure to prepare a decoction	1
Wrong decoction preparation method	2
Labelling error on decoction bottle	3
Prescribing errors	7
Prescribing the wrong medicine	3
Prescribing the wrong dose	3
Failure to discontinue medication	1
Adverse drug events	4
Kampo medicine-induced interstitial pneumonia	4

**Fig. 5:**
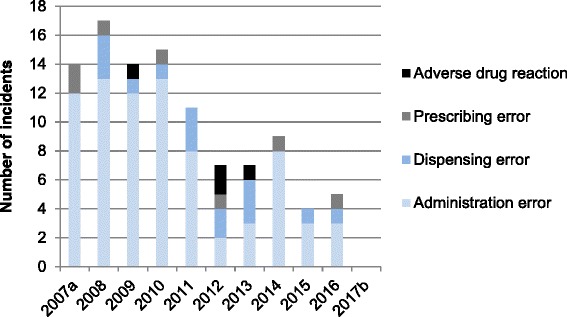
Annual numbers of Kampo medicine-related incidents. a: Data for 8 months, from May to December 2007. b: Data for 4 months, from January to April 2017

The annual numbers of incidents related to Kampo medicines were more than 10 from 2007 to 2011, but were less than 10 thereafter; in particular, the number of drug administration errors was markedly decreased (Fig. 5). Regarding the formulation of Kampo medicine that triggered the 103 incidents, 74 (71.8%) were decoctions and 29 (28.2%) were extracts.

### Severity of incidents related to Kampo medicines

We modified the severity classification made by the incident reporter for 10 of the 103 incidents because they were considered inappropriate according to the incident severity classification system recommended by the National University Hospital Council of Japan, and we needed to ensure consistency with the evaluations of the other incidents. As such, of the 103 incidents, 10 (9.7%) were classified as level 0 (an error occurred, but the patient was not affected), 89 (86.5%) were level 1 (an error occurred that affected the patient, but did not cause harm), two (1.9%) were level 2 (the patient was transiently harmed, but needed no treatment), and two (1.9%) were level 3b (the patient was transiently harmed and required substantial treatment). All 99 medication errors were classified as level 1 or less; of the four adverse drug events, two were level 2 and two were level 3b.

## Discussion

In Japan, Kampo medicines are available, not only as non-prescription drugs (i.e., over-the-counter drugs), but also as prescription drugs (i.e., ethical drugs). The application of Kampo medicines for ethical use has steadily increased, and according to a survey conducted by the Japan Kampo Medicines Manufacturers Association in 2011, 89.0% of physicians prescribed Kampo medicines in their daily medical practices [11]. The Japan Society for Oriental Medicine is the largest academic society for Kampo medicine, with 8518 regular members and 2086 certified physician members as of March 2016 [12]. In Japan, a medical license is unified and there is no specific medical license of Kampo medicine; therefore, it is possible for physicians who are not certified by the Japan Society for Oriental Medicine as Kampo medicine specialists to prescribe ethical Kampo formulations. The development of modern, ready-to-use forms of Kampo medicines has resulted in increased usage, mainly as spray-dried granular extracts of the original formulas. Kampo extracts for ethical use have largely replaced traditional decoctions of the crude drugs, even though crude drugs are also covered by the national health insurance system. Japanese physicians with limited knowledge of Kampo medicine tend to prescribe ethical Kampo extracts, not crude drugs for decoction, based on their knowledge of conventional Western medicine.

In the present survey, most incidents were drug administration errors. All of these occurred in hospital wards, involved nurses, and were not considered serious. It appeared that more incidents were associated with nurses having fewer years of experience; however, in university hospitals, nurses tend to have less professional experience than the physicians and pharmacists, and often transfer departments every few years. The annual number of administration errors tended to decrease during the 10-year period surveyed. This may be the result of improvement in the nurses’ approach to patient safety, because feedback has been given to hospital staff regarding the importance of incident reporting for the safety of patients and prevention of errors in the hospital. Another possible explanation is that the annual number of inpatients served by the Department of Japanese Oriental Medicine also decreased during this period.

In the Toyama University Hospital, the pharmaceutical department dispenses not only Kampo extract formulations, but also decoctions of Kampo medicines for inpatients. This system is rare in Japanese hospitals, especially university hospitals. In the present research, there were 15 dispensing errors reported, nine of which occurred during preparation of the decoctions and six occurred during extract preparation. Additionally, among these errors, 11 occurred during the dispensing process to inpatients, and four to outpatients. All 15 incidents were not considered serious. In recent years, out-of-hospital prescriptions have rapidly become more common in Japan, while conventional hospital prescriptions for outpatients have decreased. The ratio of out-of-hospital prescriptions for the Toyama University Hospital has also increased, being reported at approximately 90% for the fiscal year ending in March 2017. Therefore, a limitation of the present survey is that we were unable to investigate incidents that may have occurred at out-of-hospital pharmacies (i.e., community pharmacies).

Of the seven prescribing errors, three were incidents of “prescribing the wrong medicine”. All were committed by physicians who belonged to departments other than the Department of Japanese Oriental Medicine, and the erroneous drug names were similar to those of the correct medications. Thus, insufficient knowledge of Kampo medicine is a likely cause of error. Meanwhile, two of three incidents of “prescribing the wrong dose” were errors involving crude drug dosing made by physicians of the Department of Japanese Oriental Medicine, both of which were considered not serious.

All four adverse drug events discovered with the present survey were cases of drug-induced interstitial pneumonia, and were classified as adverse drug reactions. Two patients recovered after discontinuation of Kampo medicines, while the other two required hospitalization and steroid therapy. In Japan, a case of interstitial pneumonia induced by a Kampo formulation, shosaikoto (Xiao Chai Hu Tang), was reported in 1989 [13]; thereafter, a number of cases of Kampo medicine-induced interstitial pneumonia have been reported. Presently, interstitial pneumonia is listed as an adverse reaction in the package inserts of 30 types of ethical Kampo extract formulations. In the present survey, the Kampo medicine (crude drug) that was thought to induce interstitial pneumonia in all four cases was Scutellariae Radix (*Scutellariae baicalensis* Georgi), which is consistent with past reports [14–18]. The causal relationship between Scutellariae Radix and interstitial pneumonia has not been completely elucidated, but involvement of an allergic-immunological mechanism is suspected [19]. Although not observed in this survey, liver dysfunction is also listed as an adverse reaction in the package inserts of many Kampo extract formulations in Japan, and a possible close causal relationship with Scutellariae Radix is suspected [14].

## Conclusions

In the 10-year survey period (from May 2007 to April 2017), we discovered 103 Kampo medicine-related incidents from the incident reports filed in the Toyama University Hospital. Of those, 99 were medication errors (77 administration errors, 15 dispensing errors, and seven prescribing errors) and four were adverse drug events (all cases of interstitial pneumonia). These findings suggest that patient safety should be promoted to prevent medication errors, especially administration errors, and adverse drug events related to Kampo medicines.

## Abbreviation

WHO: World Health Organization.
